# Characterization of Chromosome Stability in Diploid, Polyploid and Hybrid Yeast Cells

**DOI:** 10.1371/journal.pone.0068094

**Published:** 2013-07-10

**Authors:** Rajaraman Kumaran, Shi-Yow Yang, Jun-Yi Leu

**Affiliations:** 1 Molecular Cell Biology, Taiwan International Graduate Program, Institute of Molecular Biology, Academia Sinica, and Graduate Institute of Life Sciences, National Defense Medical Center, Taipei, Taiwan; 2 Institute of Molecular Biology, Academia Sinica, Taipei, Taiwan; Florida State University, United States of America

## Abstract

Chromosome instability is a key component of cancer progression and many heritable diseases. Understanding why some chromosomes are more unstable than others could provide insight into understanding genome integrity. Here we systematically investigate the spontaneous chromosome loss for all sixteen chromosomes in *Saccharomyces cerevisiae* in order to elucidate the mechanisms underlying chromosome instability. We observed that the stability of different chromosomes varied more than 100-fold. Consistent with previous studies on artificial chromosomes, chromosome loss frequency was negatively correlated to chromosome length in *S. cerevisiae* diploids, triploids and *S. cerevisiae-S. bayanus* hybrids. Chromosome III, an equivalent of sex chromosomes in budding yeast, was found to be the most unstable chromosome among all cases examined. Moreover, similar instability was observed in chromosome III of *S. bayanus*, a species that diverged from *S. cerevisiae* about 20 million years ago, suggesting that the instability is caused by a conserved mechanism. Chromosome III was found to have a highly relaxed spindle checkpoint response in the genome. Using a plasmid stability assay, we found that differences in the centromeric sequence may explain certain aspects of chromosome instability. Our results reveal that even under normal conditions, individual chromosomes in a genome are subject to different levels of pressure in chromosome loss (or gain).

## Introduction

Chromosomes are thread-like structures made up of DNA and proteins present in the nucleus of cells and are essential for transmission of genetic information. Organisms maintain a wild type euploid complement of chromosomes that determine the properties of the cell. Therefore, the correct segregation of chromosomes between cells is of utmost importance. Chromosome segregation is a highly accurate process where mechanisms have evolved to replicate and segregate chromosomes with high fidelity [1]. To ensure the fidelity of chromosome segregation, cells employ a surveillance mechanism called the spindle checkpoint, which monitors the attachment of sister chromatids to the mitotic spindle before their separation. If sister chromatids are not properly attached to the spindle, the spindle checkpoint delays the cell cycle, allowing additional time to repair the error [2]–[4]. Nonetheless, various types of chromosome instability have been reported in many organisms [5]–[7].

Failure of proper segregation of chromosomes can have dire consequences for the cell. Mitotic chromosome loss causes aneuploidy (a change away from the euploid chromosome number), resulting in growth defects due to gene dosage imbalance [8]–[10]. Mitotically induced aneuploidy in diploid fission yeast is unstable and cells readily become haploid [11]. Except in rare cases, aneuploid plants disappear from populations after a few generations [12].

Genome instability is a common feature of human tumor cells. Although a tumor karyotype may remain quite stable over time, genome instability will cause variation, and often individual cells within a tumor will possess different karyotypes [13]. Understanding the mechanisms behind chromosome instability is important to understand how the cell maintains genome integrity and hence prevents deleterious effects including cancer formation.

Chromosome instability in yeast has been characterized in a few individual chromosomes. Chromosome VII instability was studied under normal and chemical induced conditions [14]–[17], and also in polyploid cells [18]. The stability of chromosomes X, V, and III was measured in other studies [19], [20]. It is still unclear whether different chromosomes behave similarly during mitosis or whether there are chromosome-specific factors affecting the stability of individual chromosomes.

A number of *cis*-acting elements have been shown to affect chromosome stability: origins of replication [21], the centromeres of chromosomes where spindle fibers attach to the chromosomes [22], [23], and telomeres [24]–[26]. Previous studies have suggested that chromosomal stability also depends on the size of chromosomes [27]–[29], sister chromatid cohesion [30], [31], chromosome fragile sites [32], [33], and genes involved in double-strand break repair pathways [34], [35], microtubule dynamics [36] and cell cycle checkpoints [2], [4], [37].

In this study, we report systematic measurements of the chromosome loss frequency of all sixteen chromosomes in diploid and triploid *S. cerevisiae* cells, and also in *S. cerevisiae*-*S. bayanus* hybrid diploid cells. Among sixteen chromosomes, chromosome III exhibited high instability in all three backgrounds. We carried out detailed molecular studies to dissect the mechanism behind the instability of chromosome III. Our results suggest that chromosome III is less sensitive to the spindle checkpoint, and multiple *cis*-acting elements are involved in chromosome III stability.

## Materials and Methods

### Strains and Genetic Procedures

All of the sixteen chromosomes of the lab yeast strain S288C were marked separately with *URA3* and *KanMX4* selective markers inserted into the near-centromeric regions on the left and right arms, respectively (see Table S1 for the precise location of the inserted marker). All the marked haploid lines were tested for their growth rates and only the ones that grew similarly to wild type cells were used in our chromosome loss experiments. *MATa* haploid cells of *Saccharomyces cerevisiae* with a marked chromosome were mated with α haploid or α/α diploid cells of *S. cerevisiae* to generate diploid or triploid zygotes, or were mated with α haploid cells of *S. bayanus* to generate hybrid diploid cells. The DNA content of each construct was analyzed by flow cytometry to confirm the ploidy. In some experiments, strains isogenic with *W303* (*MAT* ***a*** *ura3-1 his3-11,15 leu2-3,112 trp1-1 ade2-1 can1-100*) were also used. Substitutive and integrative transformations were carried out by the lithium acetate procedure [38]. Media, microbial and genetic techniques were performed as described [39].

### Estimation of Chromosome Loss in Diploid and Triploid Cells

Diploid or triploid zygotes were grown in CSM (Complete Supplement Mixture) -Ura medium to a similar saturation density, diluted 1000-fold in the same medium, and grown to a similar saturation density again (so all diploid and triploid cell lines have grown for a similar number of generations during this process). 1–10×10^6^ cells (determined by plating diluted cultures on YPD plates) were plated on 5-FOA (5-fluoroorotic acid) plates and plates were incubated at 30°C for one week to measure the Ura- colony number. The Ura- colonies were replica plated to YPD+G418 plates to examine whether they were G418-sensitive cells. Only colonies that had lost both *URA3* and *KanMX4* markers were counted and chromosome loss frequency was calculated by dividing the Ura- and G418-sensitive colony number to the total plated cell number. At least 5 biological replicates were performed in each measurement. To examine whether Ura- cells would divide or lose viability in CSM-Ura medium, we measured chromosome loss frequency of chromosome III from the same cultures for four consecutive days. Only after 3 days the loss frequency was observed to decline significantly, indicating that Ura- cells maintained their viability for at least 3 days in CSM-Ura (Figure S1).

### Fluctuation Analysis for Measuring Loss of the *URA3* Maker on Chromosomes III and IX

Fluctuation tests were carried out to determine the loss rate of the *URA3* marker on chromosomes III and IX. Diploid and triploid strains carrying marked chromosomes III or IX were grown in CSM-Ura medium to saturation. Cell cultures were then diluted and low numbers of cells (∼1,000) were inoculated into at least 30 independent 100 µl YPD cultures per strain in 96 well plates. Cultures were left over night at 30°C until the cultures were assessed to have reached a suitable density, and then the entire culture except for 5 µl was plated onto pre-dried 5-FOA plates. The remaining culture was used to determine the cell number. 5-FOA plates were incubated at 30°C for one week to measure the Ura- colony number. Each experiment was repeated three times. *URA3* loss rates were calculated using the maximum likelihood method via the online calculator “FALCOR” (http://www.keshavsingh.org/protocols/FALCOR.html#interface) [40], [41].

### DNA Content Analysis

5×10^6^ cells were washed in ddH_2_O, then again in an ice-cold solution comprised of 40% EtOH, 0.1 M sorbitol and 5 µM EDTA before being stored overnight at −20°C. Cells were recovered from this suspension by centrifugation, the supernatant discarded and then resuspended in 1 ml of 0.5% Triton X-100 (prepared in PBS), mixed well, centrifuged, the supernatant discarded and then resuspended in 50 mM Tris-Cl, 100 µg/ml RNAase. This solution was incubated overnight at 37°C. Staining of the DNA was achieved by adding 0.3 ml of Sytox Green Solution [1 part 5 mM Sytox Green (Invitrogen, Carlsbad, CA) to 800 parts 38 mM sodium citrate]. Stained cells were then sonicated for 3 min, diluted 1∶5 in PBS and then at least 10000 cells scored for DNA content using a FACScan (Becton-Dickinson, Franklin Lake, NJ).

### Mating Assay

In the mating assay, 2×10^6^ *MATα* cells (or diploid cells that lost the copy of chromosome III carrying *MATa*), which are Trp+ Ura-, were mixed with 2×10^7^ *MATa* cells, which are Trp- Ura+, and then spread on a 5 cm^2^ area of YPD plates. After 3.5 hours of mating at 30°C, cells were washed off and plated on CSM-Trp plates at a density of about 300 colonies/plate. In the same experiment, cells were also allowed to mate for a longer period of time (6 hours) but no obvious difference in the zygote formation frequency was observed. The number of mated cells was determined by replica plating these colonies onto CSM-Trp-Ura plates. The mating efficiency was calculated by dividing the number of mated cells to the number of *MATα* cells. At least three independent mating plates were set up and their average is shown at each sample point.

### Pulsed Field Gel Electrophoresis and Southern Blot

Karyotypes of chromosome III-lost samples were analyzed by pulsed field gel electrophoresis. A total of 1∼2×10^8^ yeast cells was used for plug preparation. Cells were washed with 1 ml EDTA/Tris (50 mM EDTA, 10 mM Tris, pH 7.5) and transferred into EDTA/Tris with 0.13 mg/ml zymolyase (Seikagaku America Inc., St. Petersburg, FL). The cell mixtures were incubated for 30 s at 42°C and then embedded in low melting point agarose (Sigma-Aldrich, St. Louis, MO). The agarose plugs were placed at 37°C overnight for zymolyase digestion. After digestion, the agarose plugs were placed in LET solution (0.5 M EDTA, 10 mM Tris, pH 7.5) containing 2 mg/ml protease K and 1% N-lauroylsarcosine at 50°C overnight. This step was repeated three times. The plugs were transferred to EDTA/Tris solution and dialyzed four times for 1 h at 37°C. Yeast chromosomes were separated on 0.7% agarose gels by pulsed field gel electrophoresis (PFGE) using a Rotaphor®Type V apparatus (Biometra, Göttingen, Germany). Electrophoresis was performed for 48 h at 13°C in 0.5× TBE buffer at a fixed voltage of 120 V and an angle of 115° with pulse time intervals of 30 sec.

After PFGE, the chromosomal DNA was depurinated and denatured by incubating the agarose gel in 0.25 N HCl and then in alkaline solution (0.5 M NaOH, 1.5% NaCl). The DNA was transferred to a charged nylon membrane, Immobilon-NY+ (Millipore, Billerica, MA). DNA probes were obtained by PCR a locus on the right arm (from 210,078 to 211,108 bp) or the left arm (from 53,372 to 54,379 bp) of chromosome III. The Digoxigenin-labeled DNA probes were prepared using a DNA labeling and detection kit (Roche, Indianapolis, IN).

### Chromosome Arm Exchange by the Cre-Lox System

A *LEU2-promoter-loxP-KanMX-loxP* cassette was inserted into the right arm of chromosome III at the intergenic region near *YCR024C* by homologous recombination. A *loxP-KanMX-loxP-promoterless-LEU2-coding region* cassette was inserted into the left arm of chromosome IX at the intergenic region near *YIL108W*. Similar constructs were generated in both strains with or without the marked chromosomes. After the constructs were confirmed, a plasmid carrying the *cre* gene driven by a *GAL1* promoter (pSH62) was introduced into the cells to pop out *KanMX* [42]. Cells carrying the *LEU2-promoter-loxP* cassette were mated with cells carrying the *loxP-promoterless-LEU2-coding region* cassette, and the Cre-induced recombination between the arms of chromosomes III and IX was performed to obtain translocated chromosomes. After the constructs were confirmed, cells were grown in YPD to lose the plasmid containing *cre* and the diploid strains were used to determine the chromosome loss frequency.

### Triple Deletions of *MAT*, *HML* and *HMR*

The *HMR* and *HML* loci were first deleted by homologous recombination using the clonNAT and hygromycin resistance markers, respectively, in the *MATa* cells carrying marked chromosome III. After the construct was confirmed, cells were then mated with *MATα* cells to generate diploid cells. The knockout of *MATa* was carried out in the diploid cells using the *MATa* knockout cassette [43].

### Plasmid Stability Assay

*CEN3, CEN9*, *CEN12*, *CEN14* and *CEN15* with 500 bp flanking sequences on both sides were PCR amplified from yeast genomic DNA and *ARS* from plasmid pRS416. PCR-annealed *ARS* and *CEN* were ligated to pRS406 to obtain CEN plasmids. Yeast cells carrying the plasmid were grown in CSM-Ura medium to saturation, diluted 1000 fold in the same medium, and grown to saturation again. 7×10^4^ cells were plated on 5-FOA plates to measure the Ura- colony number. At least 5 biological replicates were used in each measurement.

### Integration of *ARS305* at the *YCR001W* Locus

*ARS305* with 500 bp flanking sequences on both sides was amplified from yeast genomic DNA, and a hygromycin resistance gene (*hph*) was amplified from plasmid pAG32. These two fragments were fused together and inserted into the *YCR001W* locus by homologous recombination.

### Examination of *ARS305* Origin Firing at the *YCR001W* Locus

Strains with or without the insertion of *ARS305* at the *YCR001W* locus were arrested at G1 phase by treating with alpha factor at 10 µg/ml for two and a half hours. Arrested cells were then washed twice with water to remove alpha factor and resuspended in fresh medium to resume cell cycle. Samples collected at different time points were fixed, examined for their DNA content by FACS as previously mentioned, and used to isolate genomic DNA, Genomic DNA was then subjected to real-time quantitative PCR analysis to determine the copy number of a locus using locus-specific primers, the SYBR Green PCR master mix, and an ABI-7000 sequence detection system (Applied Biosystems). Data were analyzed using the built-in analysis program.

## Results

### Estimation of Chromosome Loss in *S. cerevisiae* Diploid Cells

**Figure 1:**
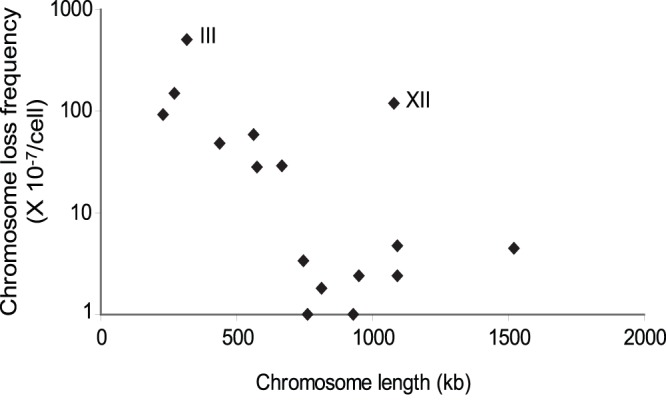
The relationship between chromosome size and chromosome loss frequency in *S. cerevisiae* diploid cells. In general, the chromosome loss frequencies negatively correlated with chromosome lengths (Spearman’s rank correlation coefficient = −0.669, *p* = 0.009). Chromosomes III and XII are two chromosomes having unusually high loss frequencies among the short and long chromosome groups.

To estimate chromosome loss, we first constructed 16 *S. cerevisiae* haploid strains (*MATa* cells) each with *URA3* and *KanMX4* selective markers inserted into the near-centromeric regions on the left and right arms, respectively (see Materials and Methods and Table S1 for details). These marked haploid strains were then crossed with a *MATα* haploid strain without the selective *URA3* and *KanMX4* markers. To estimate the chromosomal loss frequency, these diploid cells were grown in CSM-Ura media for about 40 generations and then 1–10×10^6^ cells were plated on 5-FOA plates to measure the number of colonies that had lost the *URA3* marker. During growth, cells might have become Ura- either by mutating in the *URA3* gene or losing the whole marked chromosome. These two types of cells could be distinguished by checking the *KanMX4* marker using G418 resistance on the other arm of the same chromosome. Only cells losing the whole chromosome or having a large-scale chromosomal rearrangement would be Ura- and G418 sensitive. Since the Ura- cells would not divide but would still maintain the viability in the CSM-Ura medium, each Ura- cell represented an individual mutation or chromosome loss event. Therefore, we could estimate the chromosome loss frequency by dividing the Ura- and G418-sensitive colony number to the plated cell number. We observed that the chromosome loss frequency varied more than 100-fold between different chromosomes (Table 1). In general, the chromosome loss frequencies were negatively correlated with the chromosome lengths (Spearman’s rank correlation coefficient = −0.594, *p* = 0.015; Figure 1). Nonetheless, chromosomes III and XII showed unusually high loss frequencies among the short and long chromosome groups.

**Table 1:** Chromosome loss frequencies in *S. cerevisiae* diploid, *S. cerevisiae-S. bayanus* hybrid diploid and *S. cerevisiae* triploid cells.

		Chromosome loss frequency (X 10^−7^/cell)		
Chromosome number	Chromosome size (kb)	Pure diploids	Diploid hybrids	Pure triploid
Chromosome I	230	91.8±8.3	12.0±3.6	122.0±12.6
Chromosome II	813	1.8±1.1	1.4±0.7	0.6±0.4
Chromosome III	315	504.0±58.9	732.0±143.0	226.0±58.5
Chromosome IV	1522	4.5±0.8	1.0±0.4	2.6±1.3
Chromosome V	574	28.4±3.7	12.6±2.6	13.4±4.0
Chromosome VI	270	148.0±14.2	38.0±14.3	124.0±23.5
Chromosome VII	1090	4.8±0.8	27.0±6.8	3.3±1.3
Chromosome VIII	562	58.8±10.2	25.0±8.0	52.5±0.9
Chromosome IX	439	47.8±6.4	45.2±9.4	30.2±7.0
Chromosome X	745	3.4±2.2	2.2±1.3	1.0±0.6
Chromosome XI	666	29.4±2.4	15.0±6.4	8.4±4.6
Chromosome XII	1078	119.6±7.2	2.8±0.7	40.0±13.4
Chromosome XIII	924	<0.2	<0.2	<0.2
Chromosome XIV	784	<0.2	0.8±0.2	<0.2
Chromosome XV	1091	2.4±0.5	0.4±0.4	1.0±0.4
Chromosome XVI	948	2.4±0.6	0.2±0.2	0.4±0.4

To further confirm that different chromosomes do have different loss rates, we used fluctuation tests to compare the mutation rate of *URA3* on marked chromosomes III and IX in diploid cells [41]. Our rationale is that although various mutations including point mutations, chromosomal rearrangements and chromosome loss will contribute to the observed mutation rate, chromosome loss will have a major contribution since the observed loss frequencies of chromosomes III and IX are higher than the mutation rates of other types [44]. Indeed, a significant difference (*t* test, p<0.001) was observed between the *URA3* mutation rates of chromosome III (3.0–4.3×10^−5^, 95% confidence interval) and chromosome IX (0.8–1.4×10^−5^, 95% confidence interval).

Previous studies have observed that the chromosome loss frequency was influenced by sister chromatid cohesion density and the replication origin number [21], [45]. We asked whether the observed chromosome loss frequency is correlated with the autonomously replicating sequence (*ARS*) density or cohesin density of each individual chromosome. However, no significant correlation was found (Table S2).

### Estimation of Chromosome Loss in *S. cerevisiae* Triploid and *S. cerevisiae-S. bayanus* Hybrid Diploid Cells

To determine how much chromosomal stability is influenced by cell ploidy, we crossed the marked haploid strains with an α/α diploid strain to construct triploid strains and determined the chromosome loss frequency. The chromosome loss frequencies in triploid cells were not that different from those observed in diploid cells (Table 1). Again, we used fluctuation tests to measure the mutation rate of *URA3* on marked chromosomes III and IX in triploid cells. We observed a significant difference (*t* test, p<0.001) between chromosome III (1.7–2.6×10^−5^, 95% confidence interval) and chromosome IX (0.3–0.6×10^−5^, 95% confidence interval) and the mutation rates are comparable to the observed chromosome loss frequencies.

Chromosomes in interspecific hybrid diploid cells have been shown to have higher loss rates probably due to incompatibility between two different genomes [46]. We were interested to know whether such incompatibility also existed between two related species, *S. cerevisiae* and *S. bayanus*. *S. bayanus* shares 62 and 80% intergenic and coding region nucleotide identity, respectively, with *S. cerevisiae*. The nucleotide substitution level in intergenic regions between *S. bayanus* and *S. cerevisiae* corresponds roughly to that between human and mouse [47]. About 95% of protein-coding genes in *S. bayanus* have orthologs in *S. cerevisiae* [48]. We crossed the marked *S. cerevisiae* haploid strains with a *S. bayanus MATα* strain to construct the hybrid diploid cells and measured the chromosomal loss frequency. Most chromosomes showed similar chromosomal loss frequencies as observed in *S. cerevisiae* diploid cells (Table 1).

A Spearman rank-order correlation test between chromosome loss frequencies of all sixteen chromosomes was performed to test whether the diploid, triploid and hybrid diploid backgrounds have different influences on the general chromosome behavior. A significant correlation existed between *S. cerevisiae* diploids and triploids (Spearman correlation coefficient = 0.972, p<0.01), and between *S. cerevisiae* diploids and *S. cerevisiae-S. bayanus* hybrid diploids (Spearman correlation coefficient = 0.752, p<0.01), indicating that the relative stability between most chromosomes was not affected by these backgrounds.

### Insertion Positions of the *URA3* and *KanMX4* Markers do not Affect Chromosome III and XVI Loss Frequency

To rule out the possibility that the high loss frequency of chromosome III was due to the insertion positions of *URA3* and *KanMX4*, we constructed another marked chromosome III by inserting *URA3* at *YCL009C* and *KanMX4* at *YCR004C*. We did not observe an obvious difference in chromosome III loss frequency between this newly constructed strain and the previous strain (Table 2).

**Table 2:** Insertion positions of the *URA3* and *KanMX4* markers do not affect chromosome III and XVI loss frequency.

Chromosome number	Insertion site of *URA3*	Insertion site of *KANMX4*	Chromosome loss frequency (X 10^−7^/cell)
Chromosome III	YCL001W (1903 bp; T)	YCR001W (1184 bp; A)	280.0±26.6
Chromosome III	YCL009C (8837 bp; A)	YCR004C (3183 bp; T)	292.0±23.9
Chromosome XVI	YPL001W (228 bp; T)	YPR004C (7934 bp;T)	1.16±0.19
Chromosome XVI	YPL003W (2549 bp; T)	YPR004C (7934 bp;T)	1.20±0.25

Some of our marked chromosomes have markers inserted very near to and transcribing towards the centromere (see chromosome IV, IX, XIII, XIV and XVI in Table S1). It has been shown that a strong promoter close to the centromere can compromise the centromere function [49]. Again, we examined the marker position effect by changing the *URA3* insertion site from *YPL001W* to *YPL003W* and measuring chromosome XVI loss frequency. A similar loss frequency was observed, indicating that inserting *URA3* near a centromere does not affect chromosome loss rate (Table 2).

### Chromosome III has a High Loss Rate in *S. bayanus*

Our chromosome loss experiments showed that chromosome III has the highest loss rate among all *S. cerevisiae* chromosomes. We were interested to know whether this phenomenon is *S. cerevisiae* specific. We marked chromosomes III and IX (which is also a small chromosome but has a lower loss rate in *S. cerevisiae*) in *S. bayanus* cells with *URA3* and then estimated the chromosome loss frequency in *S. bayanus* diploid and triploid cells (Table S3). The loss frequencies of these two chromosomes in *S. bayanus* were comparable to those in *S. cerevisiae*, indicating that the instability of chromosome III is not *S. cerevisiae*-specific.

### The Chromosome III-lost Lines are Aneuploid

**Figure 2:**
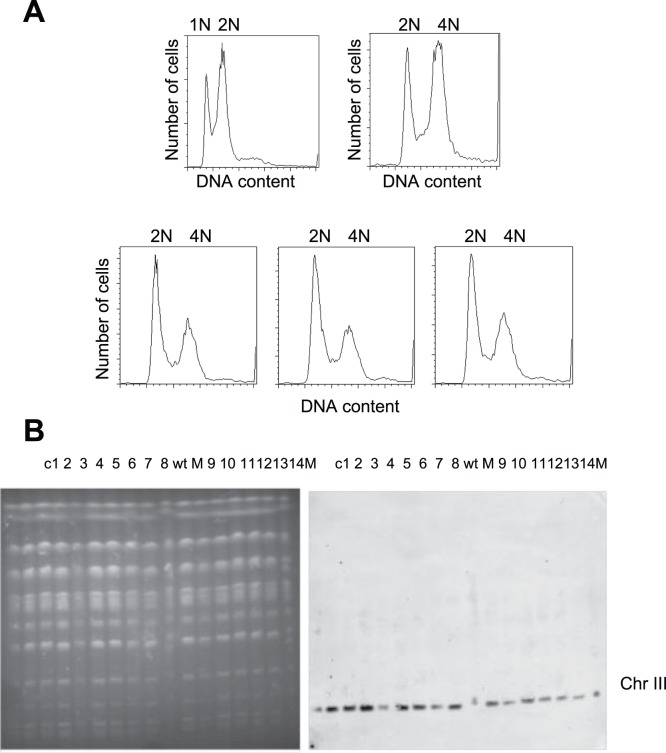
No large-scale genome rearrangements are detected in chromosome III-lost lines. (A) DNA content of chromosome III-lost lines was similar to that of diploid cells. Cells were stained with Sytox Green and analyzed by fluorescence-activated cell sorting. The first and second panels are haploid and diploid control cells, respectively. The bottom three panels show three examples of chromosome III-lost lines. (B) No obvious chromosomal rearrangements are detected in chromosome III-lost lines. The karyotypes of 14 chromosome III-lost clones (c1 to c14) were analyzed using pulsed-field gel electrophoresis. Southern blot showed hybridization using a probe from the left arm of chromosome III (from 53,372 to 54,379 bp). M, yeast chromosomal DNA marker from a standard laboratory strain; wt, wild type cells used in our experiments.

Sporulated cells or cells conducting meiosis I-like chromosome segregation will generate progeny that may not have the chromosome with *URA3* and G418-resistance markers. Two analyses were carried out to confirm that the cells that had lost the markers were really aneuploid. First, we examined the total DNA content of 10 chromosome III-lost lines using fluorescence-activated cell sorting (FACS). All chromosome III-lost clones maintained a near-diploid DNA content, suggesting that cells were not haploidized (examples shown in Figure 2A). In the second analysis, we knocked out a copy of *STE50* encoded on chromosome III in the same 10 chromosome-lost lines and examined their phenotype. The rationale is that deleting one copy of *STE50* will result in the *ste50* mutant phenotype if only one chromosome III is present in the chromosome III-lost lines. On the other hand, if cells still carry two copies of chromosome III, no obvious mutant phenotype will be revealed. It has been shown that the *ste50* mutation reduces mating efficiency drastically [50]. We mated *MATa* cells and *MATα* cells carrying two different genetic markers and measured mating efficiency by counting zygote formation (see Materials and Methods). All *ste50*-deleted chromosome III-lost cells had mating efficiencies similar to *ste50*-deleted haploid cells, suggesting that chromosome III-lost cells contain only one copy of chromosome III (Table S4).

### Chromosome III-lost Lines do not Contain Large-scale Chromosomal Rearrangements

Large-scale chromosomal rearrangements have been suggested to play a role in chromosome instability [19]. Hiraoka and colleagues observed aberrant chromosome III of various sizes due to intrachromosomal rearrangements in spontaneous loss of heterozygosity (LOH) clones. In our chromosome loss experiments, we measured chromosome loss by loss of the *URA3* marker on the left arm and *KanMX4* on the right arm of chromosomes. Our measurement of chromosome loss frequency might not be accurate if loss of *URA3* and *KanMX4* occurred due to large-scale chromosomal rearrangements. In order to investigate the conformation of individual chromosomes in the clones that have been determined by 5-FOA and G418 sensitivity, the karyotypes of these clones were analyzed using pulsed-field gel electrophoresis (PFGE). We did not detect any obvious chromosome rearrangement in all 14 lines examined (Figure 2B). Using probes from both the right and left arms of chromosome III on Southern blot of the pulsed-field gel, we further confirmed that no translocation has occurred in chromosome III (Figure 2B and data not shown).

### Multiple *cis*-acting Factors are Involved in Chromosome III Instability

**Figure 3:**
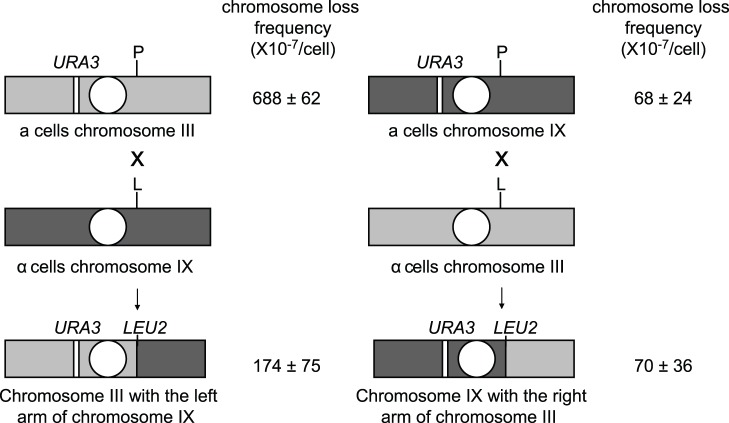
Diagram illustrating the design of chromosome arm exchange experiments and the chromosome loss frequency of different constructs. Chromosomal arms marked with *URA3* were used to measure chromosome loss frequency. White circles represent the centromere. The *promoter-loxP* (P) and *loxP-promoterless-LEU2-coding region* (L) cassettes were inserted into different chromosomes. The Cre-induced site-specific recombination between the arms of chromosomes III and XI was performed in diploid cells as described in Materials and Methods. After recombination, a functional *LEU2* gene was generated so cells could grow on CSM-Leu plates. Data represent the mean ± s.e.m. of five biological replicates. P, *promoter-loxP*; L, *loxP-promoterless-LEU2-coding region*.

Because chromosome III exhibited high instability in both *S. cerevisiae* and *S. bayanus*, we suspected that the instability might be caused by some chromosome-specific factors. In order to narrow down the region of chromosome III that is responsible for its high instability, we exchanged the right arm of chromosome III (between *YCR024C* and the telomere) with the left arm of chromosome IX (between *YIL108W* and the telomere) and then measured the loss rate of these chimeric chromosomes (Figure 3). Chromosome IX was chosen since its size is not much bigger than chromosome III, but its loss rate is much lower. Following the exchange of the similarly-sized chromosome arms (160 kb) of chromosome III and IX, both chimeric chromosomes had a lower loss rate than chromosome III. On the other hand, the stability of the hybrid chromosome III containing the left arm of chromosome IX (173.6±74.5×10^−7^/cell) was lower than the stability of chromosome IX (68.0±24.1×10^−7^/cell) and another hybrid chromosome (70.0±35.7×10^−7^/cell). This result implies that the left arm and the centromere of chromosome III contain a *cis*-acting factor affecting its stability. In addition, another *cis*-acting factor may exist on the right arm of chromosome III, but it can only influence chromosome stability when it is on the same chromosome with the first one.

The right arm of chromosome III harbors the *MAT* locus that is responsible for mating type switching in yeast. The presence of the *MAT* locus has been speculated to cause chromosomal instability due to recombination between *MAT* and *HMR* or *HML* loci [19], [51]. Interested to know whether knockout of all three mating cassettes *MAT*, *HMR* and *HML* could rescue chromosome III instability, we carried out triple deletion of *HMR*, *HML* and *MAT* from chromosome III and measured the chromosome loss frequency [43]. Cells with triple deletion of *MAT*, *HMR* and *HML* had a similar chromosome III loss rate (317.5±22.0×10^−7^/cell) as wild type cells (320.0±29.5×10^−7^/cell).

### The Centromeric Sequence Influences Chromosome Stability

Centromeres are essential chromosomal structures to which spindle microtubules bind and are necessary for the faithful segregation of chromosomes during mitosis and meiosis. The budding yeast centromere consists of approximately 125 bp DNA sequence which can be divided into three elements called CDEI, CDEII and CDEIII [52]. Previous studies showed that some point mutations in CDEI or CDEIII could have profound effects on the chromosome loss rate [23], [53]. Interestingly, a recent study indicated that yeast centromeres suffer a higher mutation rate than the rest of the genome [54]. The fact that centromeres are rapidly evolving raises the possibility that different centromeric sequences on individual chromosomes may contribute to the differences in chromosome stability. A plasmid stability assay was used to test this hypothesis. We constructed yeast centromeric plasmids containing an *ARS* element (yeast Autonomously Replicating Sequence) and the centromere with 500 bp flanking regions on both sides from chromosomes III, IX, XII, XIV or XV. The plasmid loss rates are shown in Table 3. In general, the centromeric plasmids are less stable than linear chromosomes. Nonetheless, except for the plasmid carrying *CEN 15*, the loss rate of most plasmids showed a similar trend to the loss rate of chromosomes (Pearson’s correlation coefficient *r* = 0.998, p = 0.002, excluding the *CEN 15* plasmid). These results suggest that different centromeric sequences influence chromosomal stability.

**Table 3:** Plasmid stability assay to determine the role of the centromere in chromosome stability.

Centromere of plasmid	Plasmid loss frequency (X10^−4^/cell)
*CEN3*	16.2±2.0
*CEN9*	4.0±0.3
*CEN12*	5.0±0.7
*CEN14*	2.4±0.7
*CEN15*	17.7±2.1

### Integration of an Active Replication Origin Near the Centromere does not Improve Chromosome III Stability

A previous study has shown that delaying centromere replication could result in chromosome instability due to failure in setting up bi-oriented sister centromeres [55]. *ARS308* is the replication origin nearest to the centromere of chromosome III and has been found to be inactive [56]–[58]. We hypothesized that if the instability of chromosome III is indeed caused by late replication of *CEN3*, integrating an active replication origin near *CEN3* may be able to rescue the instability. We integrated an active replication origin *ARS305* at the *YCR001W* locus and then estimated the chromosome III loss frequency. We found that replication origin integration had no effect on chromosome III stability (data not shown). To rule out the possibility that the inserted replication origin became inactive at the new site, we determined the genomic DNA copy number of *YCR001W* with or without the *ARS305* insertion at different time points of cell cycle. Quantitative PCR results showed that the *YCR001W* locus replicated more quickly after the insertion of *ARS305* (Figure S2).

### Mutations in the Spindle Checkpoint have Different Effects on Individual Chromosomes

The spindle checkpoint ensures the proper segregation of chromosomes during mitosis and meiosis [2]–[4]. Previous studies have shown that when the spindle checkpoint is impaired, different pairs of homologous chromosomes are affected differently in meiosis I chromosomal segregation [59]. Nonetheless, it is unclear whether the spindle checkpoint has different effects on individual chromosomes during mitosis. To address this issue, we measured the chromosome loss frequency in diploid cells with a *MAD2* deletion, a major component of the spindle checkpoint. As shown in Table 4, chromosome III still had the highest loss rate, but the difference between chromosome III and the other chromosomes was drastically reduced in the *mad2*Δ mutants. Our results suggest that the spindle checkpoint may have different sensitivity on different chromosomes when they are not properly attached.

**Table 4:** Comparison of chromosome loss frequency between wild type and *mad2*Δ mutant diploid cells.

Chromosome number	Chromosome loss frequency inwild type cells (× 10^−7^/cell)	Chromosome loss frequency in *mad2*Δcells (× 10^−7^/cell)	Fold change
Chromosome II	6.0±0. 9	86.5±22.7	14.4
Chromosome III	295.0±16.1	995.3±116.2	3.4
Chromosome IV	12.7±1.2	233.7±23.4	18.4
Chromosome VI	77.0±10.8	742.3±75.3	9.6
Chromosome IX	43.7±4.7	817.0±68.0	18.8
Chromosome XII	58.0±4.5	678.7±58.0	11.7

## Discussion

We have reported here the spontaneous chromosomes loss frequency of all the sixteen chromosomes in diploid and triploid cells. We confirmed that chromosome size is negatively correlated with chromosome loss [27]–[29]. These results suggested that catenation between sister chromatids plays an important role in the stability of natural chromosomes as previously suggested in yeast artificial chromosomes [28]. Since the chromosome loss rate is correlated with chromosome size and no drastic reduction in the DNA content is observed in the chromosome-lost lines, the observed loss frequency is likely to represent the measurement of individual chromosome loss events. However, we cannot rule out the possibility that compound losses (in which multiple chromosomes lose simultaneously) also occurred.

One caveat of the colony formation assay is that if loss of certain genes on a chromosome is very deleterious to the cell, the loss rate of that chromosome may be underestimated. Although we cannot completely rule out this possibility, we think that this may not be a major concern for our measurements. In our assays, we allowed the cells to proliferate for one week before counting the Ura- colonies, ensuring that only cells with extremely slow growth rates would be missed. In addition, our results from the *mad2* mutants showed that the loss rates could be increased, suggesting that it is possible to recover more chromosome-lost cells even for those “stable” chromosomes. A previous genome-wide study has also shown that chromosome III, the most unstable chromosome in our assay, in fact contains more haploinsufficient genes than other chromosomes [60].

Although the loss frequencies of an individual chromosome could vary between different yeast strains (e.g., the loss frequency of chromosome III ranged from 295.0±16.1×10^−7^/cell to 680.0±137.9×10^−7^/cell), the relative stability of different individual chromosomes exhibits a consistent pattern. A similar pattern of relative stability was also observed in diploid and triploid *S. cerevisiae* cells, and *S. cerevisiae-S. bayanus* hybrid cells with only a few exceptions. The stability of chromosome XII is significantly increased in hybrid cells. One possible explanation is that hybrid diploid cells missing *S. cerevisiae* chromosome XII might be less viable, resulting in an underestimation of the chromosome loss rate. This idea is supported by a previous study showing that *S. bayanus* chromosome XII is partially incompatible with the *S. cerevisiae* genome [61].

Surprisingly, we did not observe much of an increase in chromosome instability in triploid cells as previously reported [18]. It is unclear whether this was due to the differences of the strain background or the experimental conditions. Nonetheless, we have confirmed the chromosome loss rate using another assay (the fluctuation test). The mating locus effect was also ruled out since triploid cells heterozygous or homozygous for the mating locus have similar chromosome loss rates (Table S5). In addition, in a daily dilution experiment conducted in our lab, polyploid cells were stably maintained for several thousand generations (unpublished data), suggesting that the genome of triploid cells can be quite stable under certain growth conditions.

Chromosomes III and XII showed a much higher loss frequency compared with other chromosomes of similar sizes, suggesting other *cis*-acting elements are also involved in their instability. Chromosome XII contains the rDNA cluster which has been suggested to cause chromosome instability [62], [63]. Therefore, we further investigated the mechanism underlying the instability of chromosome III.

In diploid cells, chromosome III (approximately 315 kb) is 5 times less stable than chromosome I, the shortest chromosome in the genome (approximately 230 kb). This is unlikely to be an artifact of the lab yeast strains. Chromosome III of *S. bayanus*, a species that separated from the *S. cerevisiae* lineage about 20 million years ago, was also observed to be highly unstable, suggesting that a common mechanism is operating in both species. By exchanging chromosome arms between chromosomes III and IX, we found that multiple components are involved in the instability of chromosome III. Previous studies have suggested that intra-chromosomal rearrangements between active and silent mating loci on chromosome III contribute to chromosome instability [19], [51]. However, we found that deleting *MAT*, *HML* and *HMR* loci did not improve the stability of chromosome III. In addition, no aberrant chromosome III was detected in those chromosome III-lost clones, suggesting that intra-chromosomal rearrangements might not be the major factor influencing chromosome III instability.

The results from a plasmid stability assay showed that the centromeres from different chromosomes behave differently. Among five centromeres tested, the plasmid carrying *CEN3* exhibited a high loss rate, suggesting that *CEN3* might be an important factor contributing to the instability of chromosome III. In some plants and animals, centromeres have a high evolutionary rate probably due to meiotic drive [64]. Although such selection may not work on yeast, a recent paper has shown that the centromeres are the fastest-evolving part of the chromosome in *S. paradoxus*, a close relative of *S. cerevisiae* [54]. The fast-evolving centromeric sequences may explain some of the differences in chromosome stability.

The chromosome loss rate under the spindle checkpoint mutant background reveals an interesting pattern. The loss frequency of chromosome III, the most unstable chromosome in wild-type cells, increased only three fold with a homozygous *mad2* deletion whereas other chromosomes exhibited massive increases (10–20 fold) in chromosome loss frequency. Many originally “stable” chromosomes showed a loss rate similar to chromosome III in the *mad2* mutants. One possible explanation is that most chromosomes actually have a similar rate of kinetochore misattachment or unattachment during mitosis. However, the spindle checkpoint has different sensitivity to different individual chromosomes when their sister centromeres are not properly oriented. Our observation that different centromeres behave differently in the plasmid stability assay indirectly supports this hypothesis. It will be interesting to replace the centromere of stable chromosomes with centromere III and test the effect directly in future studies.

When the centromere-flanking regions (50 kb up- and downstream of the centromere) were surveyed, we observed that chromosome III harbors the highest number of LTRs (14 as compared to 6.06, the average LTR number of all chromosomes; Table S6). Regions enriched with LTRs are prone to ectopic mitotic recombination, a common mechanism leading to chromosome instability [65]–[68]. In addition, a genome-wide study has found that chromosome III contains more fragile sites, suggesting that ectopic recombination may be another factor contributing to chromosome III instability [69].

Chromosome III in *S. cerevisiae* is equivalent to the sex chromosome in higher eukaryotes since the mating type locus on chromosome III can determine the sex of a haploid cell. Sex chromosomes are often subjected to different selection pressure from that of other chromosomes [70]–[72]. It has been suggested that a tight linkage between the mating type locus and the centromere was selected in yeast evolution [73]. In addition, a recent study has shown that the structure of chromosome III is constantly changing in the *Saccharomycetaceae* family [74]. Interestingly, it has also been reported that the sex chromosome has a high loss rate in the pathogenic yeast *Candida albicans* [75], [76]. The big question that remains is whether the high instability of chromosome III results from (direct or indirect) selection or is simply a product of mutational drift.

## Supporting Information

Figure S1 The viability of Ura- auxotrophs in CSM-URA declines after 3 days. Diploid cells were grown to saturation as described in Materials and Methods and maintained in the same media. Chromosome loss frequency of chromosome III from the same cultures was measured for four consecutive days. Only after 3 days the loss frequency was observed to decline significantly. Data represent mean ± s.e.m of five replicates.
(EPS)

Figure S2 The *YCR001W* locus replicates more quickly after the insertion of the *ARS305* replication origin. Cells were released from cell cycle arrest (T0) and then collected after 20 and 60 minutes (T20 and T60). DNA copy numbers were determined by quantitative PCR using primers specific to the flanking regions of the original *ARS305* locus (ARS305-DNA) and the *YCR001W* locus (YCR001W-DNA). All the relative ratios were normalized to the ratio at T0. Data represent mean ± s.e.m. of three replicates.
(EPS)

Table S1 The insertion sites of *URA3* and *KANMX4* on marked chromosomes used to measure chromosome loss frequency.
(DOC)

Table S2 Correlation analysis between chromosome loss frequency (*S. cerevisiae* diploid) and chromosome cis-acting elements.
(DOC)

Table S3 Chromosome loss frequencies of chromosome III and chromosome IX in *S. bayanus* diploids and triploids.
(DOC)

Table S4 Estimation of zygote formation efficiency by the mating assay.
(DOC)

Table S5 Comparison of chromosome loss frequency between triploid cells heterozygous and homozygous for the mating locus.
(DOC)

Table S6 Number of LTR in 50 kb up and downstream regions flanking centromeres of sixteen chromosomes in *S. cerevisiae*.
(DOC)
