# Supplementary material for: Characterization of Chromosome Stability in Diploid, Polyploid and Hybrid Yeast Cells
Source: PLoS One. 2013 Jul 10;8(7):e68094. doi: 10.1371/journal.pone.0068094 (PMC3707968; doi:10.1371/journal.pone.0068094)
Supplement: Table S1 — The insertion sites of URA3 and KANMX4 on marked chromosomes used to measure chromosome loss frequency. (DOC) [file pone.0068094.s003.doc]

**Table S1. The insertion sites of *URA3* and *KANMX4* on marked chromosomes used to measure chromosome loss frequency.**

| **Chromosome number** | **Insertion site of *URA3*** | **Insertion site of *KANMX4*** |
| --- | --- | --- |
| Chromosome I | YAL002W (3934 bp; T) | YAR002C (2483 bp; T) |
| Chromosome II | YBL001C (740 bp; A) | YBR001C (618 bp; T) |
| Chromosome III | YCL001W (1903 bp; T) | YCR001W (1184 bp; A) |
| Chromosome IV | YDL001W (435 bp; T) | YDR001C (399 bp; T) |
| Chromosome V | YEL001C (1009 bp; A) | YER001W (1416 bp; A) |
| Chromosome VI | YFL003C (11353 bp; A) | YFR006W (7518 bp; A) |
| Chromosome VII | YGL002W (1753 bp; T) | YGR003W (3094 bp; A) |
| Chromosome VIII | YHL002W (1616 bp; T) | YHR003C (4325 bp; T) |
| Chromosome IX | YIL001W (148 bp; T) | YIR002C (1670 bp; T) |
| Chromosome X | YJL004C (4107 bp; A) | YJR001W (377 bp; A) |
| Chromosome XI | YKL001C (744 bp; A) | YKR003W (5135 bp; A) |
| Chromosome XII | YLL010C (20215 bp; A) | YLR001C (442 bp; T) |
| Chromosome XIII | YML001W (231 bp; T) | YMR002W (4044 bp; A) |
| Chromosome XIV | YNL001W (142 bp; T) | YNR002C (4133 bp; T) |
| Chromosome XV | YOL002C (2220 bp; A) | YOR005C (7807 bp; T) |
| Chromosome XVI | YPL001W (228 bp; T) | YPR004C (7934 bp; T) |

The number in parentheses indicates the distance between the insertion site and the centromere; T, transcription of the marker is toward the centromere; A, transcription of the marker is away from the centromere.
