# Supplementary material for: Characterization of Chromosome Stability in Diploid, Polyploid and Hybrid Yeast Cells
Source: PLoS One. 2013 Jul 10;8(7):e68094. doi: 10.1371/journal.pone.0068094 (PMC3707968; doi:10.1371/journal.pone.0068094)
Supplement: Table S2 — Correlation analysis between chromosome loss frequency (S. cerevisiae diploid) and chromosome cis-acting elements. (DOC) [file pone.0068094.s004.doc]

**Table S2. Correlation analysis between chromosome loss frequency (*S. cerevisiae* diploid) and chromosome cis-acting elements.**

| **Cis-acting element** | **Spearman’s correlation coefficient** | **P value** |
| --- | --- | --- |
| Cohesion density^a^ | - 0.294 | 0.269 |
| *ARS* density | - 0.467 | 0.068 |
| Transposon density | - 0.496 | 0.051 |
| Chromosome length | - 0.594^*^ | 0.015 |

^a^ The number of cohesin binding sites on each individual chromosome was obtained from Glynn et al., 2004 who have done genome-wide mapping of cohesin binding in budding yeast. The cohesin density was calculated by dividing the cohesin binding site number by the chromosome length.

* Spearman’s correlation coefficient was significant.
