# Supplementary material for: Characterization of Chromosome Stability in Diploid, Polyploid and Hybrid Yeast Cells
Source: PLoS One. 2013 Jul 10;8(7):e68094. doi: 10.1371/journal.pone.0068094 (PMC3707968; doi:10.1371/journal.pone.0068094)
Supplement: Table S3 — Chromosome loss frequencies of chromosome III and chromosome IX in S. bayanus diploids and triploids. (DOC) [file pone.0068094.s005.doc]

**Table S3. Chromosome loss frequencies of chromosome III and chromosome IX in *S. bayanus* diploids and triploids.**

| **Chromosome number** | **Chromosome loss frequency (x 10^-7^/cell)** |
| --- | --- |
| Chromosome III (Diploid) | 680.0 ± 137.9 |
| Chromosome IX (Diploid) | 102.0 ± 20.5 |
| Chromosome III (Triploid) | 424.0 ± 61.1 |
| Chromosome IX (Triploid) | 92.0 ± 24.1 |
