# Supplementary material for: Characterization of Chromosome Stability in Diploid, Polyploid and Hybrid Yeast Cells
Source: PLoS One. 2013 Jul 10;8(7):e68094. doi: 10.1371/journal.pone.0068094 (PMC3707968; doi:10.1371/journal.pone.0068094)
Supplement: Table S4 — Estimation of zygote formation efficiency by the mating assay. (DOC) [file pone.0068094.s006.doc]

**Table S4. Estimation of zygote formation efficiency by the mating assay.**

| **Yeast strains** | **Zygote formation frequency of *STE50* cells (%)** | **Zygote formation frequency of *ste50* cells (%)** |
| --- | --- | --- |
| Chromosome III-lost clone 1 | 82 | 0 |
| Chromosome III-lost clone 2 | 82 | 0 |
| Chromosome III-lost clone 3 | 81 | 0 |
| Chromosome III-lost clone 4 | 59 | 0 |
| Chromosome III-lost clone 5 | 89 | 0 |
| Chromosome III-lost clone 6 | 76 | 0 |
| Chromosome III-lost clone 7 | 87 | 0 |
| Chromosome III-lost clone 8 | 85 | 0 |
| Chromosome III-lost clone 9 | 68 | 0 |
| Chromosome III-lost clone 10 | 84 | 0 |
| Wild type haploid | 74 | 0 |
