# Supplementary material for: Characterization of Chromosome Stability in Diploid, Polyploid and Hybrid Yeast Cells
Source: PLoS One. 2013 Jul 10;8(7):e68094. doi: 10.1371/journal.pone.0068094 (PMC3707968; doi:10.1371/journal.pone.0068094)
Supplement: Table S5 — Comparison of chromosome loss frequency between triploid cells heterozygous and homozygous for the mating locus. (DOC) [file pone.0068094.s007.doc]

**Table S5. Comparison of chromosome loss frequency between triploid cells heterozygous and homozygous for the mating locus.**

|  | **Chromosome loss frequency (X 10^-7^/cell)** | |
| --- | --- | --- |
| **Chromosome number** | **α/α/α triploid cells** | **a/α/α triploid cells** |
| Chromosome III | 460.0 ± 52.8 | 436.0 ± 24.8 |
| Chromosome IX | 42.0 ± 9.7 | 50.0 ± 10.5 |
