# Supplementary material for: Characterization of Chromosome Stability in Diploid, Polyploid and Hybrid Yeast Cells
Source: PLoS One. 2013 Jul 10;8(7):e68094. doi: 10.1371/journal.pone.0068094 (PMC3707968; doi:10.1371/journal.pone.0068094)
Supplement: Table S6 — Number of LTR in 50 kb up and downstream regions flanking centromeres of sixteen chromosomes in S. cerevisiae. (DOC) [file pone.0068094.s008.doc]

**Table S6. Number of LTR in 50 kb up and downstream regions flanking centromeres of sixteen chromosomes in *S. cerevisiae*.**

| **Chromosome number** | **LTR number** |
| --- | --- |
| Chromosome I | 8 |
| Chromosome II | 7 |
| Chromosome III | 14 |
| Chromosome IV | 6 |
| Chromosome V | 4 |
| Chromosome VI | 10 |
| Chromosome VII | 4 |
| Chromosome VIII | 12 |
| Chromosome IX | 3 |
| Chromosome X | 8 |
| Chromosome XI | 5 |
| Chromosome XII | 2 |
| Chromosome XIII | 6 |
| Chromosome XIV | 2 |
| Chromosome XV | 4 |
| Chromosome XVI | 2 |
